# Artifacts classification and apnea events detection in neck photoplethysmography signals

**DOI:** 10.1007/s11517-022-02666-1

**Published:** 2022-10-17

**Authors:** Irene García-López, Renard Xaviero Adhi Pramono, Esther Rodriguez-Villegas

**Affiliations:** Wearable Technologies Lab, Department of Electrical and Electronic Engineering, Imperial College London, London, SW7 2BT UK

**Keywords:** Photoplethysmography (PPG), Pulse oximetry, Noise artifacts classification, Apnea detection, Sudden unexpected death in epilepsy (SUDEP)

## Abstract

The novel pulse oximetry measurement site of the neck is a promising location for multi-modal physiological monitoring. Specifically, in the context of respiratory monitoring, in which it is important to have direct information about airflow. The neck makes this possible, in contrast to common photoplethysmography (PPG) sensing sites. However, this PPG signal is susceptible to artifacts that critically impair the signal quality. To fully exploit neck PPG for reliable physiological parameters extraction and apneas monitoring, this paper aims to develop two classification algorithms for artifacts and apnea detection. Features from the time, correlogram, and frequency domains were extracted. Two SVM classifiers with RBF kernels were trained for different window (W) lengths and thresholds (Thd) of corruption. For artifacts classification, the maximum performance was attained for the parameters combination of [W = 6s-Thd= 20%], with an average accuracy= 85.84%(ACC), sensitivity= 85.43%(SE) and specificity= 86.26%(SP). For apnea detection, the model [W = 10s-Thd= 50%] maximized all the performance metrics significantly (ACC= 88.25%, SE= 89.03%, SP= 87.42%). The findings of this proof of concept are significant for denoising novel neck PPG signals, and demonstrate, for the first time, that it is possible to promptly detect apnea events from neck PPG signals in an instantaneous manner. This could make a big impact in crucial real-time applications, like devices to prevent sudden-unexpected-death-in-epilepsy (SUDEP).

## Introduction

Photoplethysmography (PPG) is an optical low-cost sensing technique that uses light at two different wavelengths (red: 660nm and infrared (IR): 940nm) to detect blood volume variations in peripheral tissues microcirculation [1]. The PPG signal appears as a sequence of periodic pulses representing the cardiac activity, from which the heart rate (HR) can be derived. Taking advantage of the differences in light absorption between oxygenated and deoxygenated blood, the peripheral saturation of oxygen in blood (SpO_2_%) can also be obtained [2].

Pulse oximetry devices employ PPG technology to continuously monitor these two physiological parameters which are useful in a variety of health contexts. They are ubiquitous in outpatient clinics, inpatient wards, intensive care units and operating theaters, specially when the patient is under general anaesthesia, to monitor alterations of vital signs which could be indicative of medical complications [3]. Pulse oximeters are likewise extensively used in the medical subfield of sleep medicine [4]. In the context of sleep apnea disorders, for example, patients suffer from respiratory arrests due to the obstruction of the upper airways or the loss of respiratory drive. Blood oxygen desaturations associated with apneic events are typically tracked with the PPG signal.

In clinical settings, the finger is considered the gold standard measurement site for pulse oximetry, due to its rich capillarity and the ease of attachment of the sensor probe. The earlobe and forehead are also other alternative sites for sensors positioning when the patient’s hands are unavailable (e.g. wounds, burns, surgery) [5]. However, outside the context of regulated medical devices, the wrist has become the most popular PPG measurement site for consumer fitness products, due to its suitability to meet the usability constraints of wearables [6].

**Fig. 1:**
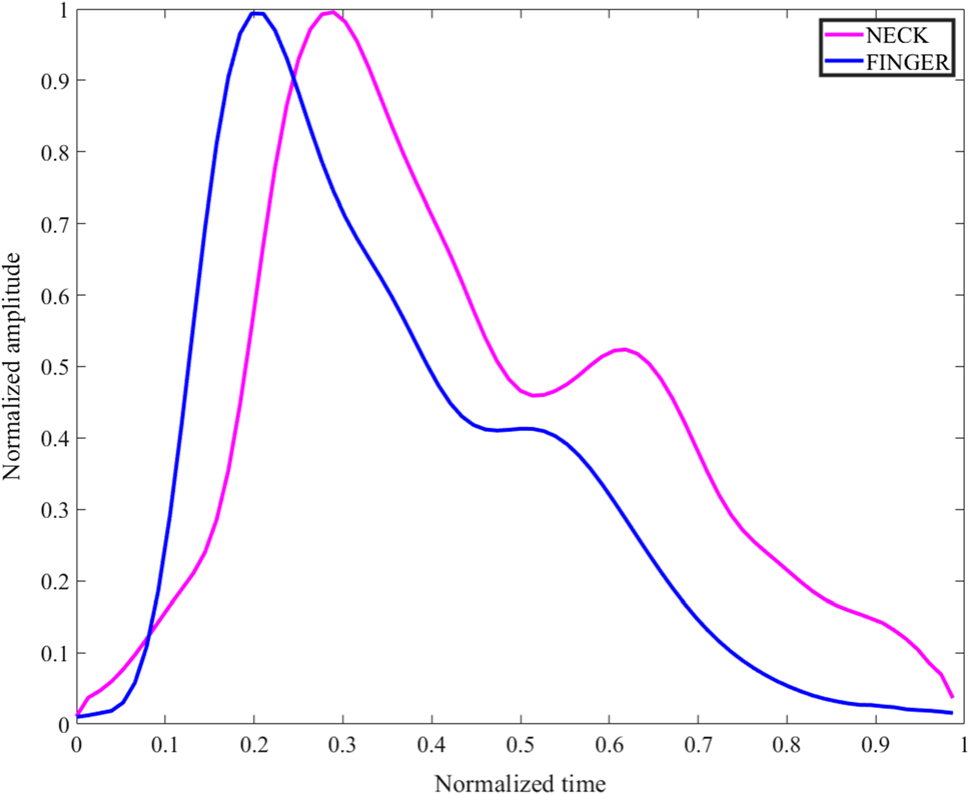
Example of neck and finger PPG pulse waveforms

The neck is a novel PPG measurement site that has not received much attention in the literature so far, but it is specially interesting for multi-modal signal acquisition. Figure 1 shows a normal PPG pulse waveform sensed from the neck and finger. The comparison of the characteristics between the two waveforms was studied in [7] where one of the findings shows that there are morphological differences between neck and finger PPG pulse waveforms such as the diastolic or dicrotic notch amplitude. Besides its comparable ability to offer access to SpO_2_% and HR biomarkers [7–9], it also offers, unlike other body parts, the unique possibility of extracting the Jugular Venous Pulse (JVP) non-invasively [10]. The neck could also provide great benefits over other conventional PPG sites in the context of some diseases for which additional physiological biomarkers are desired to be recorded simultaneously with the same wearable system. The neck for example is an exceptional location for respiratory monitoring, since airflow in the respiratory track can be sensed from it, which can be of enormous clinical value in a variety of respiratory diseases [11–13]. Specifically, for apnea detection, the neck is a unique location for cardiorespiratory multi-modal signal acquisition. In addition, we recently found that neck PPG signals were more strongly modulated by the respiratory frequency than finger PPG [7]. This makes the identification of different breathing states of interest very clear, specially when having at hand the most discriminative features [14].

In the particular case of apnea events, finger PPG pulse oximetry signals have been previously used in the literature for apnea detection together with other monitoring sensors (e.g. ECG, EEG, respiration, sound) or on their own [15–17]. Among those exclusively using PPG sensors, most of the efforts have focused on first, detecting oxygen desaturations from the surrogate SpO_2_% signal [18], and then extracting relevant apneic characteristics [19–21]. Some of the most typical features include: time series statistics of the SpO_2_ signal, the oxygen desaturation index quantifying the severity of the drop in oxygen levels by 2%, 3%, and 4% (ODI2, ODI3, ODI4), and the desaturation area under these thresholds. Deep learning was also used to directly analyse the SpO_2_ signal in [22]. Other studies, directly employed the PPG signal to extract time and frequency domain features, such as the PPG amplitude, beat-to-beat characteristics, or the low (0.04–0.15Hz) and high (0.15–0.5Hz) frequency powers [23, 24]. Papini et al. [25] included both pulse rate variability (PRV) and respiratory activity derived features from the PPG signal. Lázaro et al. [26] focused on detecting decreases in amplitude of the PPG signal (DAP) that were previously shown to be correlated with apnea [27]. However, these still depend on the detection of the delayed DAP segment of the signal occurring after the apnea. Present PPG apnea detection methods could therefore be effective in clinical scenarios, where recordings are post-processed offline. However, they show limited utility in more real-time applications. For example, in Sudden Unexpected Death in Epilepsy (SUDEP), the prompt detection of apneic events could be a matter of life or death. Neck PPG signals could offer a solution to the current limitations, as apneic respiratory arrests can be instantaneously recognized by monitoring time and frequency features [7, 14].

The acquisition of neck PPG signals is however limited by the presence of artifacts that superimpose to the signal of interest. Hence, the occurrence of head movements, coughing or swallowing could lead to unreliable and inaccurate SpO_2_ and HR readings; which in certain situations could put the patient’s life at risk, and in others could lead to discontinuous adoption due to false alarms. In order to improve the accuracy on the quantification of these physiological parameters, artifacts removal and signal reconstruction methods have been extensively developed and reported in the literature. Some include time and frequency filtering approaches like discrete wavelet transforms [28, 29], Fourier series analysis [30], predictor coefficient [31] or source separation techniques (e.g. independent component analysis [32] or singular value decomposition [33]). These approaches are, however, prone to the introduction of delays and/or distortion in the noise-free PPG segments. Adaptive filtering strategies have also been widely explored [34, 35], using additional sensors (accelerometers) to provide a noise reference estimate. Other approaches have focused, instead, on detecting and removing artifact-corrupted PPG sections, prior to the estimation of the physiological parameters of interest [36–38]. Following this approach, several machine learning algorithms have been proposed in the literature to discriminate artifacts from clean PPG. Examples of signal processing techniques used in these algorithms include: decision lists [39–43], decision trees [44, 45], naïve Bayes classifiers [46], support vector machines (SVM) [36, 47–50], multi-layered perceptrons [51], personalized neural networks (NN) [52], and 1-D CNNs [53, 54].

In the specific case of neck PPG, we have previously defined and characterized the most common neck PPG artifacts [14]. However, in our previous work and any previous research, there is no evidence of the development of algorithms for neck PPG artifacts classification. Since artifacts removal is crucial for neck PPG to work in real life conditions, the first goal of this paper was to design a high performance classifier capable of discriminating artifacts that were characterized in our previous work, from clean PPG signals. In addition, given that neck PPG signals have a big potential to instantaneously detect apneic events, the second objective of this work was to develop, for the first time in literature, an apnea classification model utilizing neck PPG.

## Methods

### Experimental protocol

In our previous work [14], a set of artifacts, including fast breathing, talking, head and body movements, swallowing, coughing, yawning and sensor rubbing, as well as two additional respiratory states of interest (slow breathing and breath-holding apnea), were recorded in a series of experiments. The study included 19 healthy participants, 12 males and 7 females, with an average BMI of 23.02 ± 2.89 *kg*/*m*^2^ and average age of 25 ± 3 years old. Written consent was obtained from all subjects and the research was approved by the Local Ethics Committee of Imperial College London (ICREC ref.: 18IC4358). Two PPG sensors were used for data acquisition in supine position: a reflectance pulse oximeter (8000R, Nonin) placed at the suprasternal notch of the neck and a transmission one (Onyx II 9560, Nonin) placed on the index finger for reference purposes. PPG signals acquisition was synchronous for both sensors at a sampling frequency of 75Hz.

This dataset was used in this paper for both artifacts classification and apnea detection. It consisted of 13 recordings per subject, of 140s duration each. During the first control recording, participants were instructed to breath at their normal respiratory pace. Then, to test other respiratory states, they were asked to modulate their respiratory frequency at three different moments in the recording for 20–30s. In one recording at a slower pace, and in another recording by holding their breaths to simulate apneic events. Ultimately, the last 10 recordings introduced different neck PPG artifacts in alternating periods of 20s with spontaneous breathing in between.

During data acquisition, the onsets and offsets of artifacts were marked in real-time. After the experiments, the annotations were verified by comparing with reference finger PPG signals. Each recording was independently normalized.

### Features extraction

#### Windows segmentation and labelling

In order to obtain relevant features for further classification, recordings were segmented in small data fragments. The extracted features were averaged within a defined time window that was repeatedly shifted by 2s along the whole recording. Each average feature corresponded to an independent observation to be inputted into the classification model. In this manner, every new upcoming bit of data was evaluated, simulating real-time processing conditions. Various window lengths (*W* = 4, 5, 6, 7, 8 and 10s) were explored to assess which one maximized the accuracy of classification.

The labelling of each window, as *artifact / clean PPG* for the artifacts classification model, or as *apnea / normal PPG* for the apnea detection model was defined based on a percentage (%) threshold *Thd* of window corruption. In other words, if let’s say *Thd* = X% or more of the evaluated PPG segment total length contained an artifact (or apnea) signal, then the window was assigned to the positive class. Otherwise, if the percentage of corruption was less than *Thd* = X%, the window was labelled as the negative class: clean PPG (or normal PPG respectively). Several thresholds of corruption (*Thd* = 20%, 30%, 40%, 50%) were tested as well to explore how the different labelling criteria affected the sensitivity and specificity of the algorithms.

#### Features

Most of the features proposed in our previous study [14], were also considered in this work, since they demonstrated strong statistical significance in the differentiation between normal clean PPG from artifacts or breathing states [14]. The time and frequency domain features were extracted to obtain the morphological beat to beat characteristics from individual or consecutive pulse segments. Meanwhile, correlogram based features were extracted since periodic pulse waves are expected to exhibit high correlation compared to artifacts which have non-periodic nature. New additional features derived from the envelope of the PPG signal were additionally included, to increase the classification performance. The 51 features considered in this study for both classification models are presented below. Further details on these features can be found in [14].

- Time domain morphological features:

**Amplitude** [*F*_1_] vertical distance between the onset of a PPG pulse and the systolic peak.

**Width** [*F*_2_] time duration between the onset and offset of a PPG pulse in time units (seconds).

** Peak Height Difference** [*F*_3_] relative amplitude between successive pulses peaks.

**Peak Distance** [*F*_4_] horizontal distance between successive pulses peaks (in seconds).

**Trough Difference** [*F*_5_] relative amplitude difference between onsets of successive pulses.

**Rise Time** [*F*_6_] time period between the onset of a PPG pulse and its systolic peak.

**Skewness** [*F*_7_] degree of symmetry of a PPG pulse.

**Kurtosis** [*F*_8_] degree of sharpness of a PPG pulse.

**Change** of *F*_1−8_ features [*F*_9−16_] instantaneous difference of feature’s values for consecutive pulses.

**Standard Deviation** of *F*_1−8_ features [*F*_17−24_] the features’ standard deviation over the whole window length.

**Zero-Crossing Rate** [*F*_25_] number of times per second that the PPG signal crosses zero.

- Correlogram features:

**Correlogram Peaks** [*F*_26-27_] autocorrelation values of the first and second peaks of the correlogram.

**Correlogram Lags** [*F*_28−29_] lags of the first and second correlogram peaks.

- *Frequency domain features:*

The one-sided modified periodogram estimate of the power spectral density (PSD) was used to calculate the frequency features. For that, the spectrogram was derived using the squared magnitude of the Short-Time Fourier Transform (STFT) with a window of 10s and 90% overlap. The output power (dB/Hz) was then sliced in time to obtain each window PSD.

**Shannon Spectral Entropy** (0–1.5Hz and 1–4Hz) [*F*_30,31_] degree of “disorder” of the power spectrum’s probability distribution.

**Spectral Kurtosis (0–1.5Hz and 1–4Hz)** [*F*_32,33_] peakedness of the PSD at each specific frequency. It is calculated as the normalized fourth-order moment of the real part of the short-time Fourier transform.

**Relative Power** [*F*_34−36_] calculated by adding the power contained within specific frequency bands (0–0.8Hz, 0.8–1.3Hz, 1.3–1.8Hz) and dividing it by the total power spanning all frequencies.

**Average Band Power** [*F*_37−41_] power of the signal was averaged within the five frequency bands: 0–0.8Hz, 0.8–1.3Hz, 1.3–1.8Hz, 2.2–2.8Hz, 3.2–3.8Hz.

- Envelope features:

The upper envelope of the PPG signal was extracted using spline interpolation over local maxima separated by at least 50 samples (> 0.667s). A total of 10 features were extracted from this envelope signal.

**Envelope standard deviation** [*F*_42_] variance in the envelope signal within the window.

**Envelope maximum** [*F*_43_] maximum value of the envelope signal within the specific window.

**Envelope minimum** [*F*_44_] minimum value of the envelope signal within the specific window.

**Envelope range** [*F*_45_] difference between the maximum and minimum values of the envelope signal within the current window.

**Envelope approximate Entropy** [*F*_46_] regularity statistic that measures the unpredictability of repetitive patterns. In other words, a PPG envelope signal including repetitive fluctuations, such as spontaneous breathing, would show small approximate entropy values, whereas a less predictable signal (e.g. artifact) would be characterized by larger ones. It was computed using the *approximateEntropy()* function in MATLAB 2020 [55].

**Envelope area** [*F*_47_] area under the envelope absolute signal, computed by numerical integration via the trapezoidal method.

**Envelope Average Power** [*F*_48−51_] power of the envelope signal was averaged within the following frequency bands: 0–0.15Hz, 0.2–0.5Hz, 0–0.5Hz and 0.5–1Hz.

**Fig. 2:**
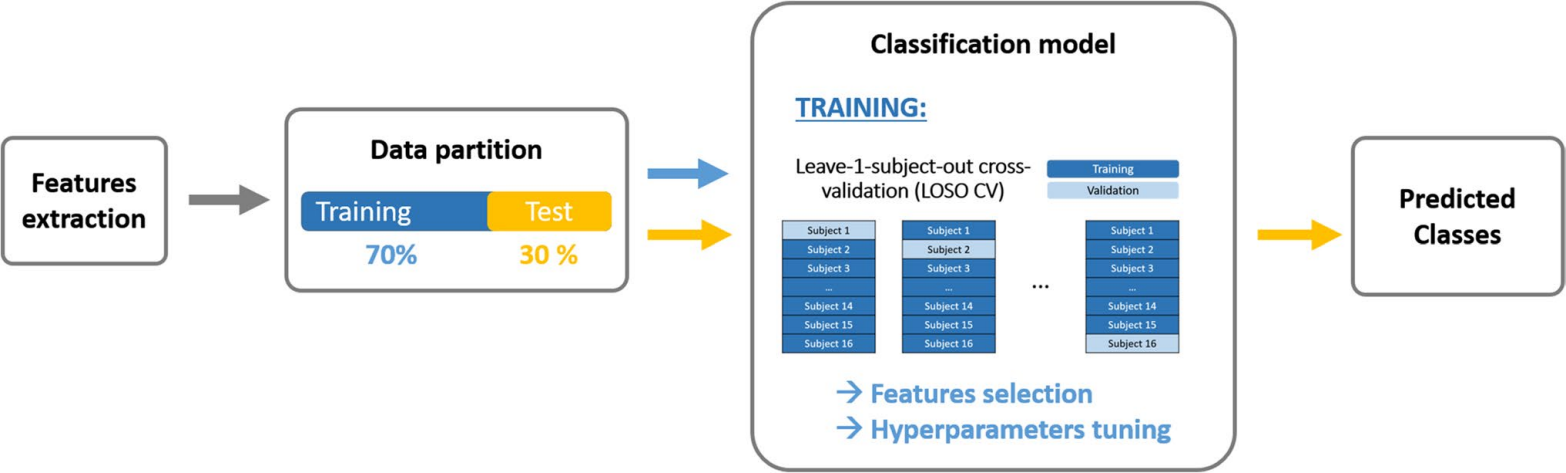
Classification pipeline. The predicted output classes for artifacts classification are: Artifacts/clean PPG; for apnea classification: Apnea/Normal PPG. This process was repeated 30 times with different randomization in the data partition stage, for each window length and threshold of corruption (%) combination

### Classification pipeline

In this study, two classification algorithms were developed: an artifacts classifier and an apnea classifier. According to our previous findings [14], on the one hand, neck PPG artifacts, with similar noisy characteristics, could be clearly distinguished from normal PPG. On the other hand, normal, slow breathing and apnea PPG signals shared common stable clean PPG features. As a consequence, for the artifacts classifier, all the artifact types were grouped together under the *artifacts* positive class; while the negative *clean PPG* class encompassed: the normal, apnea and slow breathing PPG signals.

**Fig. 3:**
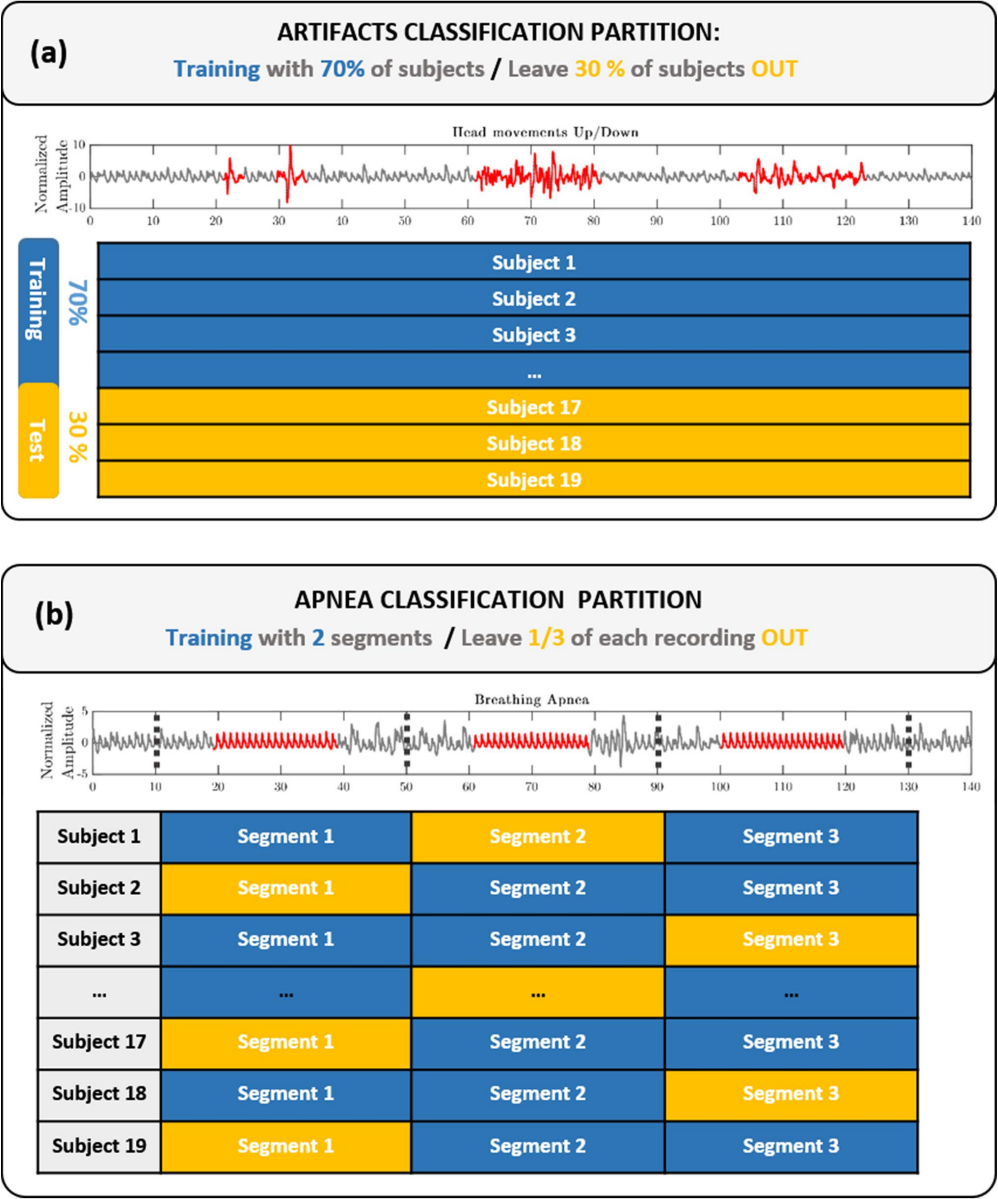
Data partitions for artifacts classification and apnea detection models. (**a**) Leave-30%-of-Subjects-Out approach for artifacts classification. (**b**) Leave-1/3-of-Recording-Out per subject for apnea detection

In order to detect apneic events among the clean PPG signals category, an apnea classifier was also engineered. The positive class consisted of the *apnea* PPG signals. And the *normal PPG* negative class comprised the normal and slow breathing categories. The number of artifacts and breathing states were evenly sampled at random in order to perform balanced binary classification.

Figure 2 shows an overview of the classification pipeline for both classifiers. This process was repeated 30 times with different randomization in the data partition stage, for each combination of window length and threshold of corruption (%). Each stage is further detailed in the subsections below.

#### Data partition

Since there was window overlapping, a random partition of data could no longer be used, as the condition of independence between training and test data would be violated.

As it can be observed in Fig. 3, two types of data partitions were used for classification. For artifacts classification, a Leave-30%-of-Subjects-Out approach was implemented. As Fig. 3(a) shows, for every seed, 70% of the subjects were selected at random for training (with all the recordings), and the other 30% were left for testing. This ensured that the classifier was tested against completely unseen data, which is one of the most strict validation strategies. All subjects were assigned to the test set evenly, at least 7 times each. This avoided any subject-bias.

For apnea classification, due to a limited number of breath-holding recordings, an alternative Leave-1/3-of-Recording-Out per subject partition was adopted instead. An illustration of three intercalated breath-holding events that simulate apneic events can be observed in Fig. 3(b). Apnea recordings were thus divided in three even segments for each subject. The same number of normal and apnea PPG windows were included in each of them and no overlapping windows (in the border) were allocated to either of the neighbouring segments. This prevented overfitting and guaranteed independence of the training and test sets. For each random seed repetition, one of the three segments was selected for the test set and the remaining two were used for training.

This data partition step was repeated 30 times for both classifiers, with different randomization of the training and test sets, to verify that the proposed algorithms showed a good generalization performance.

#### Training

A SVM classifier with a radial basis function (RBF) kernel was chosen for the artifacts and apnea classification. The objective of the SVM classification problem was to find the weights vector $\overrightarrow{w}$ and bias term *b* defining the optimal hyperplane, that maximizes the margin between classes and minimizes the loss term such that:

1 $$\min\limits_{w,b,\xi}\frac12\overrightarrow w^T\overrightarrow w+C\sum\limits_{i=1}^n\xi_i$$

subjected to the condition:

2 $$\min y_i(\overrightarrow w^T\phi(\overrightarrow x_i)+b)\geq1-\xi_i,\xi_i\geq0$$

where $\overrightarrow{x_i}$ are the training vectors, *y*_*i*_ the classes labels [-1,1] and *ξ*_*i*_ the slack variables. *C* corresponds to the regularization parameter that controls the trade-off between maximizing the margin ($C \rightarrow 0$) and minimizing the penalty term ($C\rightarrow ~\infty$). The function *ϕ* maps the training vectors into a higher dimensional space in order to gain linear separation. The RBF gaussian kernel used was defined such that:

3 $$K({\overrightarrow x}_i,\;{\overrightarrow x}_j)=\phi({\overrightarrow x}_i)^T\phi({\overrightarrow x}_j)=\exp(-\gamma\left\|{\overrightarrow x}_i-\overrightarrow xj\right\|)$$

where $\gamma =\frac {1}{2\sigma ^{2}}$ is the inverse of the radius of influence of the samples selected by the model as support vectors.

During training, the best features and hyperparameters, that optimized the model’s performance, were selected using the Leave-One-Subject-Out Cross-Validation (LOSO-CV) strategy. Similarly to k-fold cross-validation, the training data was repeatedly split, by selecting one subject at a time for testing, and the rest of the subjects for training. This approach avoids overfitting and prevents subject bias during feature selection and hyperparameters optimization.

#### Features selection

The features selection step was included within the LOSO-CV and was performed only on the training subjects. It consisted of two stages. First, the total 51 features were ranked using chi-square tests. These evaluated whether the features were independent of the classes labels, and then ranked the features based upon the output p-values. A small p-value revealed that the corresponding feature was dependent on the response variable, and therefore, was an important feature to consider for classification.

The top 30 features ranked with the Chi-square tests were fed into a forward sequential feature selection algorithm. In a wrapper fashion, the subsequent ranked features were sequentially added to the top 30 candidate set until the addition of further features did not decrease the average misclassification error by more than a relative tolerance of 1e-6. 
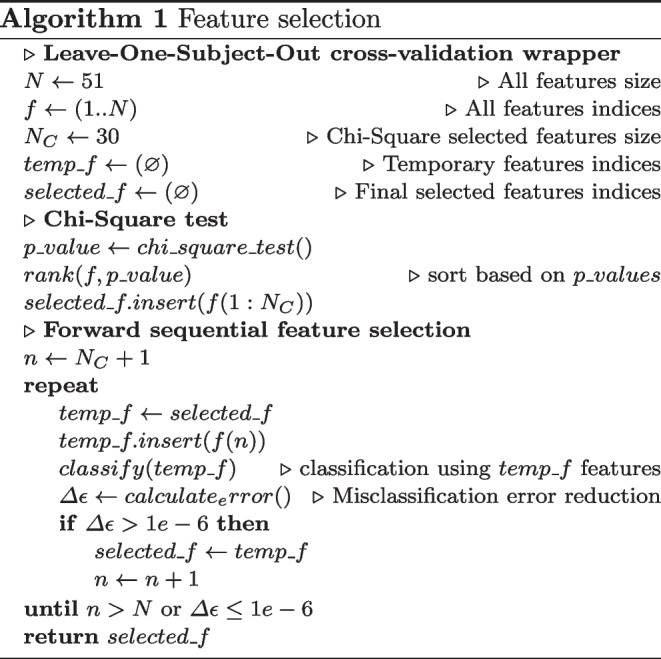


#### Hyperparameters optimization

In order to boost the SVM training performance, the soft-margin misclassification cost (*C*) and the RBF kernel gamma (*γ*) hyperparameters were optimized by grid search. For the different classifiers, all the combinations of *C* and *γ*, listed as follows, were evaluated using LOSO-CV.

$$\begin{array}{@{}rcl@{}} \mathbf{Artifacts~classification:} && C=0.5, 1, 4, 6, 8, 16, 32, 64, 80, 128\\ && \gamma=2^{-15}, 2^{-13}, 2^{-11}, ... , 2^{-1}, 2^{1}, 2^{3} \\ \end{array}$$

$$\begin{array}{@{}rcl@{}} \mathbf{Apnea~classification:} && C=0.125,0.75,1,2,3,4,5,6,8,32 \\ && \gamma=2^{-15}, 2^{-13}, 2^{-11}, ... , 2^{-1}, 2^{1}, 2^{3} \\ \end{array}$$

The hyperparameters that maximized the cross-validation training accuracy were selected for the artifacts classifier, and those showing the highest F1-score were chosen for the apnea classifier.

#### Performance metrics and model selection

Once the most optimal hyperparameters and features were selected through LOSO-CV, the final SVM model was trained with the whole training data partition. Subsequently, it was evaluated on the independent test set (in yellow in Fig. 2), to output the predicted classes.

In order to assess the classification performance of both classifiers, the following metrics (in %) were calculated as the average over the 30 randomization repetitions: accuracy (ACC), sensitivity (SE), specificity (SP), precision, and F1-score (F1).

The best artifacts classification model was chosen based on the combination of window length and threshold of corruption (%) (*W*/*Thd*) that maximized the accuracy metric. In apnea classification, the harmonic mean of precision and recall, i.e. the F1-score, was used instead to select the best *W*/*Thd* model. Indeed, the F1 metric is more relevant in this case, as the Type I (false positives) and Type II (false negatives) errors are crucial for safety in critical apnea detection applications.

### Statistical evaluation of the classification results

In order to assess whether the different windows and corruption thresholds (%) had an effect on the classification performance of both classifiers, a two-way ANOVA statistical test was carried out for each performance metric. The normality and homoscedasticity assumptions were verified using Lilliefors and Levene’s tests. This confirmed the homogeneity of variance among different sample groups and the Gaussianity of the distributions. Post hoc multiple comparisons, based on the Tukey’s honest significant difference criterion, were subsequently performed in order to investigate which pairs of means were significantly distinct, for the different windows and corruption thresholds (%) evaluated.

## Results

### Classification results

**Fig. 4:**
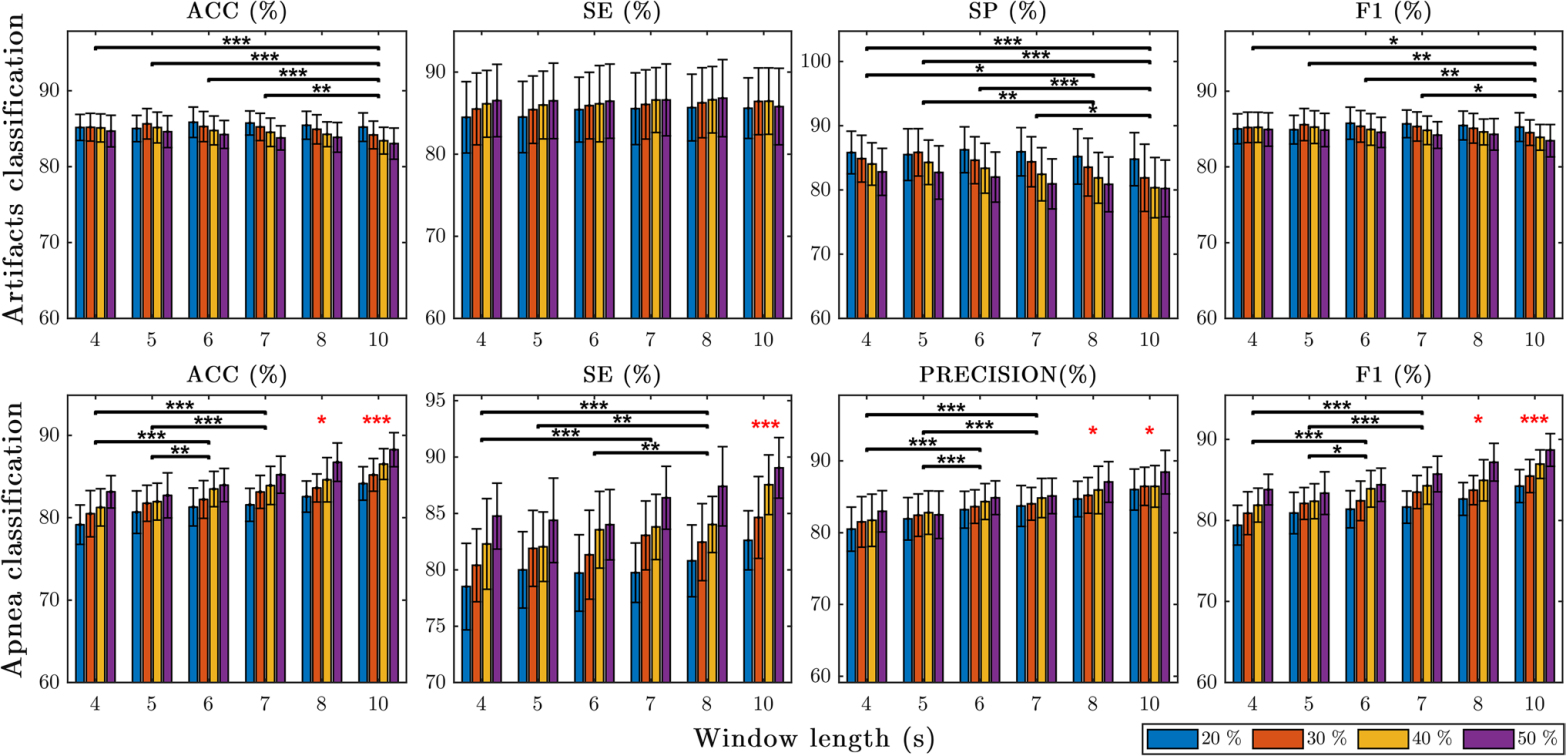
Average classification results for the proposed artifacts and apnea classification algorithms, over the 30 randomization experiments. Bar graphs show the average performance metrics across the different windows and corruption thresholds (%) tested. The error bars represent the extent of the standard deviation above and below the mean. Different thresholds of corruption (*Thd* = 20*%*, 30*%*, 40*%*, 50*%*) are specified as separate coloured bars for each window length (*W* = 4,5,6,7,8,10*s*). The statistical results of the multiple pairwise comparisons testing for the window effect are displayed with a horizontal line and a black asterisk symbol indicating the alpha significance level: * *p* < 0.05, ** *p* < 0.01, *** *p* < 0.001. The red asterisks on top of some window groups indicate that all the multiple comparisons were statistically significant for that specific window

Figure 4 shows the average results for both artifacts and apnea classification algorithms, across all windows and thresholds of corruption (%). The bar graphs represent the mean performance over the 30 repetitions and the error bars, the corresponding standard deviations. Overall, both classifiers demonstrated good performance with average metrics’ values larger than 80*%* for the majority of the *W*/*Thd* models. A more exhaustive analysis is presented in the following subsections.

#### Artifacts classification

The results presented in the upper panels of Fig. 4 show a good performance of around 86*%* for the various windows and thresholds. The *ACC*, *SE* and *F*1 mean values oscillate in a short range of 2–3% for the different *W*/*Thd* combinations. However, the *SP* mean values expand across a larger range of 6*%*, probably due to a threshold effect. On average, the standard deviations for *ACC* and *F*1 are very small (1.8%), whereas for *SE* and *SP* are slightly higher (4%). But still, these values remain acceptable considering that a Leave-30%-of-Subjects-Out validation approach was used, which is one of the most strict ones.

Table 1 presents the average performance results for the best (*W*/*Thd*) artifacts classification model. The window and corruption threshold (%) combination that maximized the accuracy of artifacts classification was *W* = 6*s* − *Thd* = 20*%*, with a value of 85.84 ± 2.00%. The *F*1-score (85.77 ± 2.12%), *SP* (86.26 ± 3.57%), and *precision* (86.29 ± 2.92%) values of this *W*/*Thd* model were also the largest compared to all other parameters pairs.

**Table 1:** Average performance results (*μ* ± *σ*, n = 30) for the best artifacts and apnea classification models

	Artifacts* classification	Apnea** classification
Best W/Thd model	W = 6s - Thd= 20%	W = 10s - Thd= 50%
True positives	1468.48 ± 63.66	131.26 ± 7.81
True negatives	1481.06 ± 67.44	120.48 ± 9.13
False positives	235.80 ± 60.74	17.42 ± 5.52
False negatives	250.84 ± 69.06	16.16 ± 4.03
ACC	85.84 ± 2.00	88.25 ± 2.07
SE	85.43 ± 3.95	89.03 ± 2.69
SP	86.26 ± 3.57	87.42 ± 3.63
Precision	86.29 ± 2.92	88.42 ± 3.04
F1	85.77 ± 2.12	88.68 ± 2.01

* The artifacts classifier discriminates between noise-corrupted PPG segments and clean data (normal breathing, slow breathing and apnea PPG fragments)

** The apnea classifier distinguishes apnea events from the rest of clean PPG data (normal breathing, slow breathing)

#### Apnea classification

In the lower panels of Fig. 4 are exposed the average classification results for the apnea classification algorithms. Although the various metrics demonstrated a good performance of around 83-84*%* in average for all the *W*/*Thd*, there was a clear ascending trend that reasonably increased the range of mean values. The difference between extreme values could span from an 8*%* in *precision* and up to a 10.5*%* in *SE*. This suggested that the windows and thresholds parameters might have had an effect. The standard deviations, pictured as error bars, occur in general very small (< 3.2*%*) for all metrics.

The best apnea classification model (*W*/*Thd*) and the corresponding performance metrics are listed in Table 1. The maximum F1 score of 88.68 ± 2.01% was obtained for the apnea classification model with a window of *W* = 10*s* and a threshold of corruption of *Thd* = 50*%*. This *W*/*Thd* combination also maximized the *ACC* (88.25 ± 2.07%), *SE* (89.03 ± 2.69%), *SP* (87.42 ± 3.63%) and *precision* (88.42 ± 3.04%), compared to the other *W*/*Thd* pairs.

**Fig. 5:**
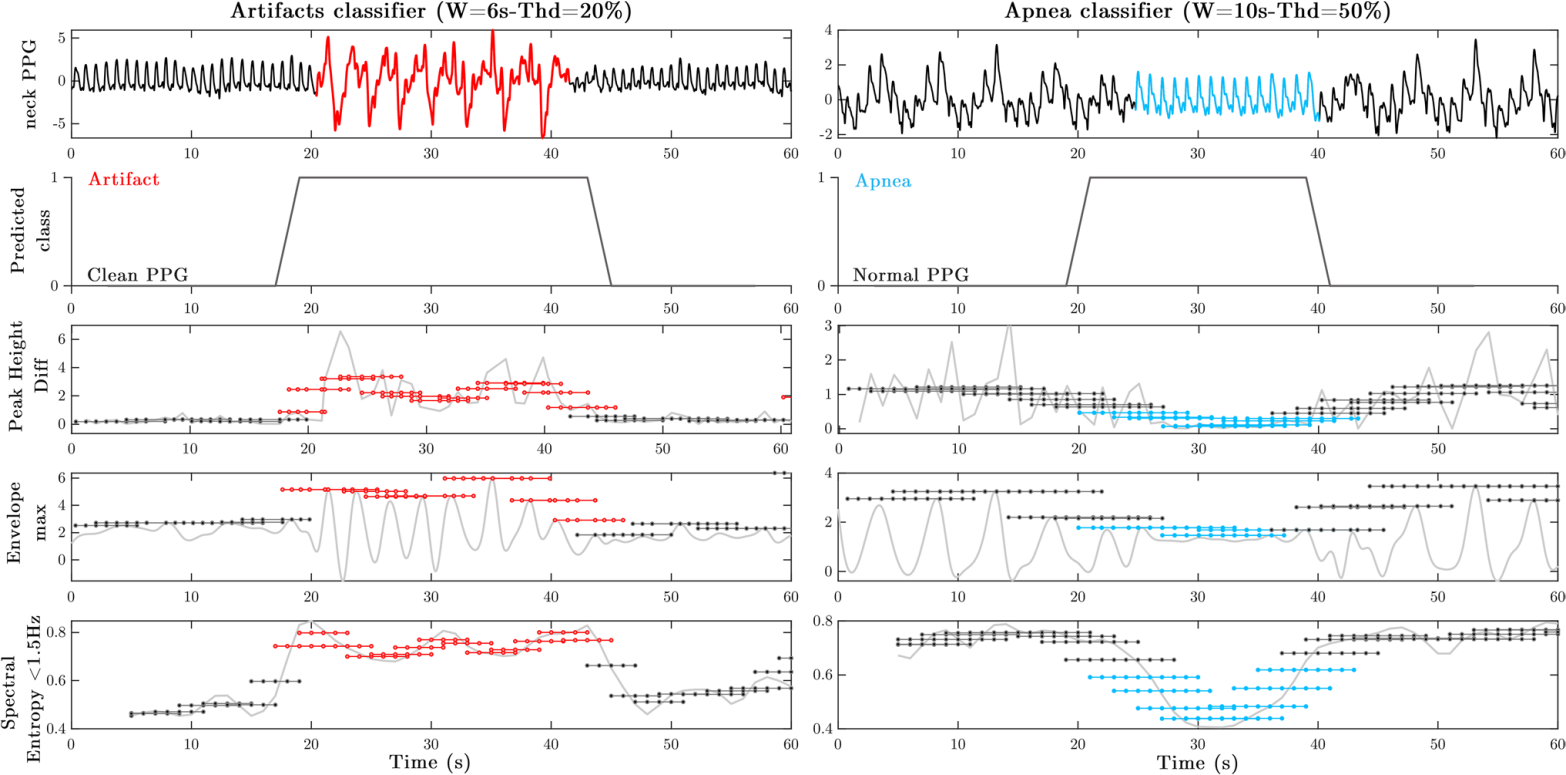
Classification decision results of the best models for one head movement artifact and an apnea event. The variation of some of the features used are displayed in the lower panels: Peak Height Difference, the maximum of the envelope and the Spectral Entropy (< 1.5Hz). True artifacts and apnea windows are labelled in red and blue respectively

Figure 5 shows the predicted classes output of the best artifacts and apnea classification models. Some of the most characteristic features that were inputted into the classifiers are also displayed, such as the Peak Height Difference, the Envelope’s maximum value and the Spectral Entropy (< 1.5Hz).

### Statistical tests results:

#### Two-way ANOVA

Overall, the resulting ANOVA tables for both classifiers, showed that the window length and the threshold of corruption (%) affected the classification performance metrics significantly (*p* < 0.05). Some exceptions to this were the window length effect for the sensitivity of artifacts classification (*p* = 0.707) and the threshold effect (%) for the specificity of apnea classification (*p* = 0.065). No statistical evidence of an interaction effect between the two factors was shown for any metric (*p* > 0.05).

The results of the post hoc multiple comparisons for the *W* and *Thd* effects are described in the next subsections.

#### Window length effect

In Fig. 4, the statistically significant pairwise differences among window lengths groups (*W* = 4,5,6,7,8,10*s*) are shown in the form of horizontal lines with an asterisk symbol representing the p-values ranges (* 0.01 < *p* < 0.05, ** 0.001 < *p* < 0.01 and *** *p* < 0.001). For the sake of visualization, a unique red asterisk symbol was used when any group was statistically significant with all the others simultaneously. The largest p-value was chosen for the asterisk representation.

As it can be observed, in **artifacts classification**, the window *W* = 10*s* shows the greatest significance. Indeed, for the average *ACC*, *SP* and *F*1 metrics, *W* = 10*s* is the only group that is statistically different from all the rest of the windows (except from *W* = 8*s*). For *SP*, besides *W* = 10*s*, the average specificity values of *W* = 8*s* are also statistically distinct from the *W* = 4 and 5*s* ones. This could be explained by a slight decrease in performance, from *W* = 5–6*s*, with increasing window length of *ACC* (-1.1*%*), *SP* (-2.7*%*) and *F*1 (-0.87*%*). No significant pairwise comparisons appear among window groups for *SE*, since, according to the ANOVA findings, the window length did not have an effect (*p* > 0.05). Actually, no dissimilarity in the average *SE* values is noticeable among window groups, being all roughly equal to 86*%* in average. The fact that the standard deviations of $\sim 4\%$ are some of the largest compared to other performance metrics, might also explain the non-significance.

In **apnea classification**, the lower panels of Fig. 4 show that the overall performance increases with longer window lengths. A rise of $\sim 5\%$ in the window means can be noticed throughout from *W* = 4*s* to *W* = 10*s*. This is corroborated with the average results of W = 10s and W = 8s being statistically distinct from the shorter windows’ lengths groups. In addition, the pairwise differences between *W* = 6,7*s* and *W* = 4,5*s* are statistically significant for the *ACC*, *precision* and *F*1 values. In the case of *SE*, the average values of the *W* = 8*s* window are also statistically distinct from the W = 4,5,6s lengths (*p* < 0.01), as well as *W* = 7*s* is different from *W* = 4*s* (*p* < 0.001).

#### Threshold of corruption (%) effect

**Fig. 6:**
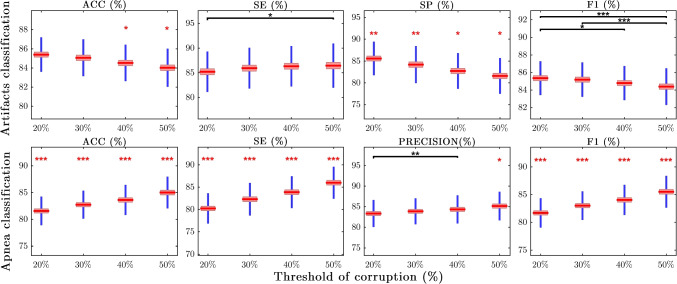
Means plots of the classification performance metrics across different thresholds of corruption (%). The means, with the corresponding 95% confidence intervals, are represented in red. Standard deviations above and below the mean are shown in blue. The statistical results of the multiple pairwise comparisons testing for the threshold effect are displayed with a horizontal line and a black asterisk for different alpha significance levels: * *p* < 0.05, ** *p* < 0.01 *** *p* < 0.001. The red asterisks on top of some threshold groups indicate that all the multiple comparisons were statistically significant for that specific window

Figure 6 shows the means plots of the classification performance metrics across different thresholds of corruption (%), for both artifacts and apnea classifiers. The statistical pairwise differences between various thresholds values (*Thd* = 20*%*, 30*%*, 40*%*, 50*%*) were displayed with asterisks as in Fig. 4.

In **artifacts classification**, it can be observed that the mean *ACC*, *SP* and *F*1 decrease with increasing percentage of corruption threshold (%), whereas the opposite happens for *SE*. The drop in average *ACC* and *F*1 performance from *Thd* = 20*%* to Thd = 50*%* is very subtle (1–2*%*), whereas for *SP* it is a bit more meaningful with a 4*%* reduction. Indeed, the mean specificity values for all the *Thd* groups are statistically distinct from one another, with a p-value of *p* < 0.01 for *Thd* = 20,30*%* and *p* < 0.05 for the *Thd* = 40,50*%* groups. For the other performance metrics (*ACC*, *SE* and *F*1), due to the small changes in mean differences among groups, only the most extreme thresholds appear to be statistically different. In fact, the pair *Thd* = 20*%*-*Thd* = 50*%* accumulates the largest number of statistically significant differences overall, followed by *Thd* = 20*%*-*Thd* = 40*%*.

In **apnea classification**, the performance metrics’ average values increased significantly with the threshold of corruption (%). The increment for *ACC*, *SE* and *F*1, was of around 5% from *Thd* = 20*%* to *Thd* = 50*%*. The mean values of all the thresholds groups for these metrics were statistically different from one another (*p* < 0.001). The mean *precision* value for *Thd* = 50*%* was also statistically significant (*p* < 0.05) with respect to the rest of the threshold groups. However, the gain in *precision* from *Thd* = 20*%* to *Thd* = 50*%* was only of 2%.

### Features selection results

**Fig. 7:**
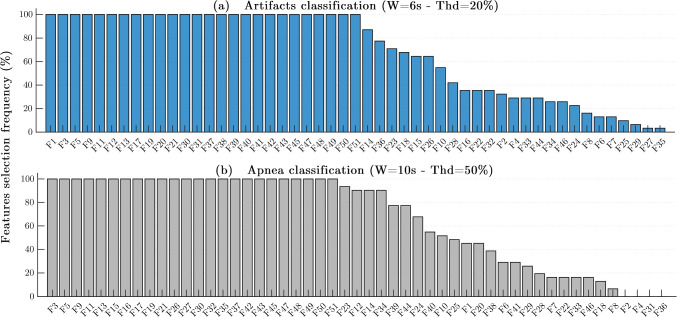
Features selection ranked by frequency of occurrence over the 30 randomization experiments for the best artifacts and apnea classification models. *F*_1_ = Amplitude, *F*_2_ = Width, *F*_3_ = PeakHeightDiff, *F*_4_ = PeakDistance, *F*_5_ = TroughDiff, *F*_6_ = RiseTime, *F*_7_ = Skewness, *F*_8_ = Kurtosis, *F*_9_ = ChangeAmplitude, *F*_10_ = ChangeWidth, *F*_11_ = ChangePeakHeightDiff, *F*_12_ = ChangePeakDistance, *F*_13_ = ChangeTroughDiff, *F*_14_ = ChangeRiseTime, *F*_15_ = ChangeSkewness, *F*_16_ = ChangeKurtosis, *F*_17_ = StdAmplitude, *F*_18_ = StdWidth, *F*_19_ = StdPeakHeightDiff, *F*_20_ = StdPeakDistance, *F*_21_ = StdTroughDiff, *F*_22_ = StdRiseTime, *F*_23_ = StdSkewness, *F*_24_ = StdKurtosis, *F*_25_ = ZeroCrossingRate (ZCR), *F*_26_ = CorrelogramPeak1, *F*_27_ = CorrelogramPeak2, *F*_28_ = CorrelogramLag1, *F*_29_ = CorrelogramLag2, *F*_30_ = SpectralEntropy (0–1.5Hz), *F*_31_ = SpectralEntropy (1–4Hz), *F*_32_ = SpectralKurtosis (0–1.5Hz), *F*_33_ = SpectralKurtosis (1–4Hz), *F*_34_ = RelativePower (0–0.8Hz), *F*_35_ = RelativePower (0.8–1.3Hz), *F*_36_ = RelativePower (1.3–1.8Hz), *F*_37_ = AvgPower (0–0.8Hz), *F*_38_ = AvgPower (0.8–1.3Hz), *F*_39_ = AvgPower (1.3–1.8Hz), *F*_40_ = AvgPower (2.2–2.8Hz), *F*_41_ = AvgPower (3.2–3.8Hz), *F*_42_ = EnvelopeStd, *F*_43_ = EnvelopeMax, *F*_44_ = EnvelopeMin, *F*_45_ = EnvelopeRange, *F*_46_ = EnvelopeApproxEntropy, *F*_47_ = EnvelopeArea, *F*_48_ = EnvelopeAvgPower (0–0.15Hz), *F*_49_ = EnvelopeAvgPower (0.2–0.5Hz), *F*_50_ = EnvelopeAvgPower (0–0.5Hz), *F*_51_ = EnvelopeAvgPower (0.5–1Hz)

Figure 7 displays the features selection frequency of occurrence (%) over the 30 randomization experiments, for the best artifacts and apnea classification models. The features were ranked in decreasing order. The most relevant features for each classification task were likely to be selected 100% of the times, while the most irrelevant ones were never chosen for the final model in any of the 30 repetitions (0%).

For **artifacts classification**, in the upper panel of Fig. 7(a), a total of 26 features were selected 100% of the times, out of the 30 repetitions. Some examples are the *Amplitude*, *PeakHeightDiff*, *TroughtDiff* and the corresponding *Changes* and *Standard deviations* of these. In the frequency domain, the *AvgPower* and *Spectral Entropy* features for all the specified bands were also some of the most important. In addition the *Envelope* characteristics were likewise predominantly selected. An additional set of 7 features that were chosen more than 50% of the times showed good discriminative potential. But, the 18 lowest ranked features, appeared less than (30%) of the times in the final classification model.

In the lower panel of Fig. 7(b), it can be observed that a set of 24 features was selected in all randomization experiments (100%) for **apnea classification**. These mainly included time domain vertical characteristics of the signal (e.g. *PeakHeightDiff*, *ThroughDiff* ), as well as the *Changes* and *StandardDeviations* of these features. All the *Envelope* characteristics (except *approxEntropy*) and the *Correlogram peaks*, were also part of the most highly selected features. In the frequency domain, the *AvgPower (0–0.8Hz)*, *RelPower (0.8–1.3Hz)*, *Spectral Entropy (< 1.5Hz)* and *Spectral Kurtosis (< 1.5Hz)* were also some of the most important features to consider for apnea detection. Besides the top (100%) features, another extra 9 were also significantly chosen more than 50% of the times. Among the rest of the 18 features selected in less than half of the 30 repetitions, the *Pulse Width*, *PeakDistance*, *SpectralEntropy (1–4Hz)* and *RelPower (1.3–1.8Hz)* were never picked for apnea classification.

## Discussion

In this paper, two automated algorithms were developed to classify noise artifacts and detect apneic events from novel neck PPG signals. A total of 51 features from the time, correlogram and frequency domains were extracted to fit both classifiers. These included morphological, statistical, and envelope characteristics of the PPG signal, as well as PSD-derived features. A SVM classifier with a RBF kernel was trained for different windows (*W* = 4, 5, 6, 7, 8 and 10s) and thresholds of corruption (*Thd* = 20%, 30%, 40%, 50%). A LOSO-CV strategy was implemented to protect against overfitting and subject bias, during features selection and hyperparameters optimization. The classifiers were tested in unseen data, to predict whether each PPG window belonged to the *artifacts*/*clean PPG* classes; and whether within the clean PPG category, it was an *apnea*/*normal PPG* segment. This process was repeated 30 times with different randomizations of the data in order to evaluate the generalization capability of the models. Overall, the results demonstrated a good average performance for both classifiers ($\sim 86\%$). The standard deviations for the different (*W*/*Thd*) models were also small enough ($\sim 2\%$) to suggest that the algorithms were very stable and could generalize well across data. This increases the confidence that the results obtained could be reliably replicated in the future, with a similar range of values, no matter the data partition. Specially, for the artifacts’ algorithm that is tested in a totally independent set of subjects (Leave-30%-of-Subjects-Out partition), the low variance indicates that the method is robust. However, some substantial differences in the performance metrics were observed among several (*W*/*Thd*) models.

The analysis of the features selected for the best (*W*/*Thd*) classification models indicated that overall, there was a recurrent set of features for each classifier, with a high chance ($\sim 100\%$) of being chosen. This suggested that features like *PeakHeightDiff* and *TroughtDiff*, as well as the corresponding *Changes* and *Standard deviations* of these, had a higher discriminative potential. The final set of features, also included *Envelope*, *AvgPower* and *Spectral Entropy* characteristics for specific frequency bands. However, around 18 features out of the total 51, were not selected many times (or even none), implying that they were not very informative for classification. The presented ranking of features offers, at hand, the most promising set of features for neck PPG artifacts classification and apnea detection. This analysis would be relevant for future studies aiming at processing neck PPG signals and improving the current classification results. It could likewise be a good starting point for additional feature engineering in related neck PPG applications.

For the **artifacts classification** results, the best *W*/*Thd* model, with the largest average accuracy (85.84 ± 2*%*), was *W* = 6*s* − *Thd* = 20*%*. This model also maximized all the other performance metrics, except for *SE* which did not show statistical significance. Even though there is a decrease in performance from *W* = 6*s* with increasing window length, the *W* = 6*s* window group only appeared to be statistically distinct from *W* = 10*s* in terms of *ACC*, *SP* and *F*1; and from *W* = 8*s* in terms of *SP*. Therefore, it cannot be straightforwardly concluded that in general, *W* = 6*s* is the most optimal window length for neck PPG artifacts classification. But, since *W* = 4,5,6 and 7*s* are statistically equally valid, and *W* = 6*s* slightly improves the overall performance, it would still be preferable to pick *W* = 6*s* as the most suitable window for future algorithms. Indeed, other PPG studies have also found appropriate window lengths in a similar range for their proposed artifacts classifiers [36, 51, 52].

In terms of threshold of corruption (%), the classification performance decreased with larger *Thd* values. Specially, the average *SP* for the optimal *Thd* = 20*%* was statistically larger than the rest of groups, hence increasing the *ACC* and *F*1 too. This suggests that, in future works, a smaller threshold of corruption for window labelling, would considerably benefit the performance of the algorithm. However, if in turn, *SE* is deemed more important, a model with larger *Thd* > 20*%* would be recommended instead.

Comparing these results with other artifacts classification studies in the literature, leads to the conclusion that our algorithm performed well. Indeed, as it can be observed in Table 2, our model showed similar *ACC*, *SE* and *SP* than the SVM classifier proposed by Couceiro [48], the decision tree by Sukor [45] or the adaptive thresholding approach by Cherif [56]. However, some algorithms exploiting fine tuned decision lists (Fischer [40]), personalized neural networks (Tabei [52]), or a linear SVM with major voting (Chong [36]), outperformed our results. But, these are just for reference and are not straightforwardly comparable because each classification problem is distinct. The measurement sites in other works are different and consequently are susceptible to different types of artifacts. Different works also implement different validation strategies.

**Table 2:** Comparison of artifacts classification results in the literature with our best (W = 6s-Thd= 20%) model

	ACC (%)	SE (%)	SP (%)
Our method	*85.8 ± 1.65*	*83.8 ± 4.1*	*87.43 ± 3.7*
Couceiro [48]	87.5 ± 0.6	78.4 ± 1.2	94.4 ± 0.6
Chong [36]	93.9	94.3	92.4
Sukor [45]	83 ± 11	89 ± 10	77 ± 19
Tabei [52]	98.07 ± 2.02	92.6 ± 6.54	99.78 ± 0.93
Cherif [56]	83 ± 8	84 ± 16	83 ± 12
Fischer [40]	98.3	99.6	90.5

The findings of this artifacts classification model, are of great importance for denoising and conditioning novel neck PPG signals, and hence, enable the possibility of exploiting this novel PPG measurement site for physiological monitoring. The removal of PPG-corrupted sections, would significantly improve the accuracy of HR and SpO_2_ readings of neck pulse oximeter sensors. Ameliorating the quality of neck PPG signals, would similarly facilitate the accurate derivation of other biomarkers of interest.

In **apnea classification**, the average performance increased with the window length and the threshold of corruption (%) by a considerable amount (> 5*%*), reaching its maximum at *W* = 10*s* − *Thd* = 50*%*. In addition, both the *W* = 10*s* window and the *Thd* = 50*%* threshold effects were shown to be statistically significant with respect to the other windows’ and thresholds’ groups for all the performance metrics. Therefore, it can be inferred that the *W* = 10*s* − *Thd* = 50*%* parameter’s combination is the most suitable for detecting apnea events with neck PPG, as it maximizes not only the F1-score (88.68 ± 2.01%), but all the other performance metrics too (*ACC* = 88.25*%*, *SE* = 89.03*%*, *SP* = 87.42*%*, *precision* = 88.42*%*).

Since *W* = 10*s* and *Thd* = 50*%* are the largest values in the ranges explored, in future studies the grid search bounds of the window length and threshold (%) parameters could be even expanded to investigate whether the performance could potentially improve. However, even though the choice of longer windows could benefit the detection, the reason behind proposing neck PPG signals as an alternative to common approaches, was to reduce the latency of other apnea detection methods. So, increasing the window length to 30s or 1min segments, would limit the utility of the proposed method for real-time applications. To illustrate, in the context of SUDEP, a longer window processing duration could increment the risk of mortality, as terminal apneas might not be that promptly detected.

**Table 3:** Comparison of apnea classification results in the literature with our best (W = 10s-Thd= 50%) model

	Signals used	ACC (%)	SE (%)	SP (%)	Precision (%)
Our method	*neck* *PPG*	*88.25 ± 2.07*	*89.03 ± 2.69*	*87.42 ± 3.63*	*88.42 ± 3.04*
Knorr-Chung [23]	PPG	75.4	91.6	84.7	85.9
Lázaro [26]	PPG	70.37	81.82	68.57	−
Papini [25]	PPG	86	39	94	51
Jung [18]	SpO_2_	91	83	89	−
Deviaene [20]	SpO_2_	82.8	64.3	88.6	64.2
Deviaene [24]	PPG+ SpO_2_	83.4	73.7	86.6	64.8

Reviewing other apnea detection approaches in the literature, the proposed RBF SVM model exploiting time and frequency characteristics directly derived from the PPG signal, outperformed both the studies exclusively extracting PPG features and the ones relying on the surrogate SpO_2_ time series. As observed in Table 3, the *SE* and *precision* values of the SpO_2_-based algorithms proposed by Deviaene et al. are poor [20, 24]. In these approaches, features extraction focused on the signal segment corresponding to the oxygen desaturation, which is delayed from the actual respiratory apnea onset by 20–40s. This lag could be critical for real-time applications. The same issue applied to the work by Jung et al. [18]. Even though they claimed to accomplish real-time apnea detection by locating the original apneic event in the preceding 25 seconds prior to the onset of the desaturation; they first had to detect the lagged response of the SpO_2_. Other SpO_2_-based algorithms in the literature, which performed epoch-based classification with window lengths of 1min or larger [57, 58], were likewise not suitable for real-time implementations.

Among the PPG works, the linear discriminant classifier proposed by Lázaro et al. [26], evaluating pulse rate variability (PRV) features from 4 windows preceding, following and spanning the delayed decreases in amplitude (DAP) events, also suffered from the same limitation. Papini et al. [25] achieved the highest specificity (*SP*) by inputting PPG-derived PRV and respiratory features into a deep learning model, but the *SE* and *precision* were insufficient for robust online monitoring. The results obtained by Knorr-Chung et al. [23], with an ANN trained on PPG time and frequency characteristics, were good but the classification model was not implemented in an epoch-by-epoch online manner. Instead, the most representative PPG fragments showing normal breathing and apneic patterns, were manually segmented for classification.

This work, in contrast, is a significant advancement in the field, since it demonstrates, for the first time in literature, that it is possible to robustly detect apnea events from neck PPG signals in an instantaneous manner, with a sliding window of 10s shifted every 2s. This is because directly detecting apnea events from neck PPG signal removed the inherent lag that would otherwise result if waiting for apnea events to translate into drops of SpO_2_, and the use of a short sliding window would mean an earlier decision can be made. The proposed method has the advantage of being simple and has the potential to be used for near real-time applications for which time lags could have a critical outcome. It could for example have a great impact in the development of monitoring systems for SUDEP prevention, by supporting airflow measurements in the decision of apnea classification. Future work should perform further experiment verification to fully validate the potential of the proposed method to be implemented as a real-time apnea detection system. Similarly, in offline applications like sleep apnea diagnosis, neck PPG signals could be of great interest for researchers as well. Not only the proposed location-specific PPG signal characteristics could be directly employed to recognize apneas; but also the SpO_2_ surrogate signal could be additionally derived, to exploit the delayed desaturation. This could hence, increase the pool of biomarkers to improve the classification performance. Moreover, the large number of cumbersome polysomnography sensors could be reduced to a unique wearable neck PPG device, capable of measuring airflow simultaneously with additional sensing modalities integrated in the same system. Future work should then focus on combining complementary respiratory signals [16], to support the classification decision and enhance the sensitivity. Tracheal sounds, for example, can be easily sensed from the multipurpose site of the neck [13, 59].

Overall, the methods in this work present useful recommendations for future designers of neck PPG processing algorithms, in terms of suggested features, window lengths, labelling thresholds and classification models. This is important for future adoption of the neck as a PPG site. Indeed, the proposed artifacts classification algorithm presents the first proof-of-concept classifier for neck PPG artifacts removal. However, once the corrupted PPG fragments are identified, a decision on how to process them should be taken. This study was devised with the idea that corrupted fragments could just be discarded, to improve the accuracy of HR and SpO_2_ parameters estimation. It does not tackle, however, the reconstruction of detected artifact signals. This should be explored in future work, specially when artifacts are expected to be predominant. Another limitation of this study is that the proposed classification models were trained using experimental artifacts or breath-holding events. These need to be tested in real sleep scenarios to validate their performance. Also, a wider number of participants, including patients prone to have apneas should be recruited. Indeed, the majority of studies developing apnea detection algorithms in the literature, make use of polysomnography databases, with apneas of different kinds (obstructive, central, mixed and hypoapneas). The accuracy of the current apnea algorithm, would probably be impacted when tested against this variety of respiratory events. Future work should then validate the current algorithms under these circumstances and potentially extract new features for the detection of less severe, shallow breathing, hypopnea events. Other machine learning techniques including deep learning could also be explored to potentially improve the performance of the proposed method when more data is available. It is important to note however that in improving efficacy, the complexity of the method should be kept to a minimum. Further improvements of this proof of concept could then ideally lead to the implementation of these classifiers in a wearable neck apnea monitoring system for apnea detection.

## Conclusion

In order to fully exploit the novel PPG measurement site of the neck, specifically to support real-time apnea detection applications, corrupted PPG segments need to be first recognized for removal. Two automatic algorithms were designed in this work to achieve these. The first classifier demonstrated good performance in distinguishing neck PPG-corrupted segments from clean PPG data; and the second showed a promising capability of promptly detecting apneic events, in a near real-time manner, both uniquely exploiting time and frequency PPG features. The preliminary results of this study, provide useful tools to facilitate neck PPG signals processing, that could encourage the future usage of the neck as a new promising PPG measurement site.
